# Whole Slide Imaging Based Digital Pathology Network between Pakistan and USA

**DOI:** 10.1155/2014/136838

**Published:** 2014-12-15

**Authors:** Syeda Fatima Absar, Mohammad Tahir, Yukako Yagi, David Wilbur

**Affiliations:** ^1^Department of Global Health and Population, Harvard School of Public Health, Boston, MA 02115, USA; ^2^Department of Pathology, Federal Government Poly Clinic Hospital, Islamabad 44000, Pakistan; ^3^Department of Pathology, Massachusetts General Hospital, Boston, MA 02114, USA

## Background

In 2007, a modern digital pathology facility was conceived at the Telemedicine Center of the Holy Family Hospital (HFH), Rawalpindi, Pakistan, in collaboration with the Pathology Department of the Massachusetts General Hospital (MGH), Boston, USA [1]. A pathologist from HFH was trained at MGH and was provided equipment with the relevant software installed. Between December 2011 and August 2012, weekly hour-long teleconferences were held between the two centers, where static histopathology images were discussed using PowerPoint. The following month, Pakistan's first whole slide imaging (WSI) scanner was installed at the Telemedicine Center at HFH; however, before it could become functional, the pathologist left the country. The aim of the project had been for MGH to provide digital pathology support to HFH and other facilities in the country; however, the untimely exit of the trained pathologist had led to a breach in communication and a hiatus in the activities. In early 2013, we reestablished the connection, utilizing the Telemedicine Center but this time with pathologists from the Federal Government Poly Clinic (FGPC) Hospital, Islamabad. Due to the needs of teleconsultation in Pakistan, we have changed the protocol to organize teleconsultation to fit the current situation.

## Method

To date, eight histopathology cases have been discussed. These cases were from tissues that ranged from the genitourinary system, central nervous system, gastrointestinal system, and soft tissue areas. At present, a consultant histopathologist and his team of four trainee pathologists from FGPC Hospital are the participants from Pakistan's side. The pertinent case slides are sent to HFH three days ahead of time by hand. They are scanned using the Panoramic Desk (40x objective lens version), 3D Histech Ltd., Hungary, as a slide scanner. Each slide takes approximately thirty minutes to be scanned. One to two slides are scanned per case. When slides are ready for discussion, a coordinator at MGH is notified via email, who then arranges a teleconference via “GoToMeeting,” Citrix Online, CA, USA. During the conference, the screen controls are handed over to HFH, presenting the case and displaying the related WSI using the MIRAX viewer. The pathologist from MGH discusses the case and captures screenshots of the relevant areas using his desktop. The Telemedicine Center at HFH has provided a network server IP address to MGH for accessing the WSI storage database at any time.

## Result

The teams conducted their first teleconference in September 2013. Subsequently, hourly meetings are held as per the requirement of FGPC, where 3-4 cases are studied. Out of the eight cases that have been discussed, the pathologist at MGH was in agreement with the FGPC diagnosis for two of the cases. His diagnosis was the same for three of the cases but after consultation with other subspecialists at MGH, he suggested a different diagnosis for one case. For two of the cases, he suggested further immunohistochemical staining. At the local front, the scanner is being used at the weekly multidisciplinary meetings at HFH. These are attended by physicians from other facilities, including the surgical, histopathology, radiology, oncology, and nuclear medicine departments at HFH, FGPC Hospital, Nuclear Medicine Oncology and Radiotherapy Institute (NORI), and the Capital Development Authority (CDA) Hospital, Islamabad.

## Conclusion

As the groundwork for teleconferencing had already been laid, all that was required was to bridge the communication gap to coordinate the activities between both centers. In this phase, we are focusing more on utilizing the WSI scanner in both local and online meetings. These discussions are a learning opportunity for pathologists and trainees at Pakistan's side and expose the USA side to rare cases. Although there is no current funding for the project, it has been successful due to the mutual interest and dedication shown by both sides. We are now able to establish regular teleconferences using WSI and our aim is to increase their frequency as required.
